# Evaluation of Microbial Bacterial and Fungal Diversity in Cerebrospinal Fluid Shunt Infection

**DOI:** 10.1371/journal.pone.0083229

**Published:** 2014-01-08

**Authors:** Tamara D. Simon, Christopher E. Pope, Samuel R. Browd, Jeffrey G. Ojemann, Jay Riva-Cambrin, Nicole Mayer-Hamblett, Margaret Rosenfeld, Danielle M. Zerr, Lucas Hoffman

**Affiliations:** 1 Department of Pediatrics, University of Washington/Seattle Children’s Hospital, Seattle, Washington, United States of America; 2 Center for Clinical and Translational Research, Seattle Children’s Research Institute, Seattle, Washington, United States of America; 3 Department of Neurological Surgery, University of Washington/Seattle Children’s Hospital, Seattle, Washington, United States of America; 4 Division of Pediatric Neurosurgery, Primary Children’s Medical Center, Department of Neurosurgery, University of Utah, Salt Lake City, Utah, United States of America; 5 Department of Microbiology, University of Washington, Seattle, Washington, United States of America; 6 Center for Infection and Prematurity Research, Seattle Children’s Research Institute, Seattle, Washington, United States of America; University of Houston, United States of America

## Abstract

**Background:**

Cerebrospinal fluid shunt infection can be recalcitrant. Recurrence is common despite appropriate therapy for the pathogens identified by culture. Improved diagnostic and therapeutic approaches are required, and culture-independent molecular approaches to cerebrospinal fluid shunt infections have not been described.

**Objectives:**

To identify the bacteria and fungi present in cerebrospinal fluid from children with cerebrospinal fluid shunt infection using a high-throughput sequencing approach, and to compare those results to those from negative controls and conventional culture.

**Methods:**

This descriptive study included eight children ≤18 years old undergoing treatment for culture-identified cerebrospinal fluid shunt infection. After routine aerobic culture of each cerebrospinal fluid sample, deoxyribonucleic acid (DNA) extraction was followed by amplification of the bacterial 16S rRNA gene and the fungal ITS DNA region tag-encoded FLX-Titanium amplicon pyrosequencing and microbial phylogenetic analysis.

**Results:**

The microbiota analyses for the initial cerebrospinal fluid samples from all eight infections identified a variety of bacteria and fungi, many of which did not grow in conventional culture. Detection by conventional culture did not predict the relative abundance of an organism by pyrosequencing, but in all cases, at least one bacterial taxon was detected by both conventional culture and pyrosequencing. Individual bacterial species fluctuated in relative abundance but remained above the limits of detection during infection treatment.

**Conclusions:**

Numerous bacterial and fungal organisms were detected in these cerebrospinal fluid shunt infections, even during and after treatment, indicating diverse and recalcitrant shunt microbiota. In evaluating cerebrospinal fluid shunt infection, fungal and anaerobic bacterial cultures should be considered in addition to aerobic bacterial cultures, and culture-independent approaches offer a promising alternative diagnostic approach. More effective treatment of cerebrospinal fluid shunt infections is needed to reduce unacceptably high rates of reinfection, and this work suggests that one effective strategy may be reduction of the diverse microbiota present in infection.

## Introduction

Cerebrospinal fluid (CSF) shunt placement has been the mainstay of treatment for hydrocephalus for over 50 years. [1] While CSF shunts allow children to survive and avoid further brain injury, they can cause new and often chronic surgical and medical problems. CSF shunt infections are one such complication, resulting in up to 2,400 pediatric hospital admissions each year in the U.S. [2] CSF shunt infections, usually associated with bacterial pathogens but occasionally fungi, [3], [4], [5], [6], [7] are notoriously difficult to treat, and adequate therapy generally requires both medical and surgical management. Medical treatment commonly involves prolonged intravenous, and occasionally intrathecal, antibiotic administration. [6], [8] However, the duration of systemic antibiotic use varies widely [8] and depends, in part, on both the surgical approach used [4], [9] and the pathogen involved. [6], [10] Surgical treatment includes a minimum of two surgeries to remove and replace the infected CSF shunt, [5], [6], [11] either shunt externalization followed by new shunt insertion once CSF cultures are negative, or shunt removal and external ventricular drain insertion followed by new shunt insertion. Published studies of the effectiveness of surgical approaches are often anecdotal in nature [12], [13], [14], [15], [16] with inconclusive results [17], [18].

For children with first CSF shunt infection, prognosis for clearance is poor, with re-infection rates ranging from 12–26 percent. [8], [9], [18] Re-infection rates are higher still for children with their second CSF shunt infection. [19] Diagnosis of CSF shunt infection currently relies on the recovery of a pathogen from conventional microbiologic cultures of CSF, and length of antibiotic treatment is often determined based on the number of days that cultures remain positive. CSF shunt replacement generally does not occur until negative CSF cultures are obtained and prescribed treatment is complete. In the majority (70%) of re-infections, the organism(s) recovered by culture are different from those recovered at first infection [19].

This recalcitrance of CSF shunt infections to treatment may be related to complex, adherent assemblages of microbes, known as biofilms, on the shunt catheter surface. [20], [21] Biofilm-dwelling bacteria grown *in vitro* exist in a largely dormant, antibiotic-resistant mode, [22] thought to account for the remarkable persistence of chronic infections despite antibiotic therapy. [23] Conventional culture techniques are designed to examine free-floating, clonal populations of a single microbial species during logarithmic growth phase. [24] These techniques do not reflect the susceptibilities of infectious biofilms, often comprised of multiple microbial taxa held together by an extracellular matrix. [25] There is no consensus for how to best grow and select antibiotics for biofilm infections, and thus traditional (liquid or agar) cultures of suspended microbial cells remain the accepted mode for diagnosing them and testing the susceptibilities of causative agents, including in the setting of CSF shunt infection. In addition, while the presence of bacteria during any infection has traditionally been determined by growth in conventional culture, [24], [26] many bacteria are difficult or impossible to cultivate, [27] and so culture-independent molecular approaches to bacterial identification are increasingly being used. [28] Culture-independent molecular approaches often identify more organisms present in chronic infection than culture. [24], [29], [30] However, before adapting newer molecular approaches for use in clinical care, it is critical to assess both the abilities of molecular methods to detect bacteria in specimens by formal comparison to conventional culture, and the clinical relevance of these methods’ more extensive results [24].

Given the recalcitrant nature of CSF shunt infection, the emerging evidence of a role for biofilms, and the increased application of culture-independent molecular approaches to medical microbiology, we sought to better characterize the diversity and natural history of the microbial communities (i.e., the microbiota) in CSF shunt infections using molecular approaches. [24] We hypothesized that culture-independent, molecular approaches would identify microbes in the CSF from children with CSF shunt infection including and beyond those pathogens identified by routine culture. The test this hypothesis, we used high-throughput sequencing to identify the bacteria and fungi present in CSF from eight children with CSF shunt infection and compared those results to similarly-analyzed negative controls as well as findings from conventional culture.

## Materials and Methods

### Ethics Statement

The study received Institutional Review Board approval from the Seattle Children’s Research Institute and the University of Utah, as well as approval from the Primary Children’s Medical Center (PCMC) Privacy Board. For all study subjects, except those from PCMC prior to March 18, 2010, consent was obtained for additional CSF to be collected on each occasion that regular CSF samples were obtained during treatment for CSF shunt infection. Prior to March 18, 2010 at PCMC, we used CSF remaining for after routine processing and testing in the PCMC Microbiology Laboratory.

### Inclusion Criteria

Children ≤18 years old undergoing treatment for conventional culture-confirmed CSF shunt infection at either Seattle Children’s Hospital (SCH) or PCMC were eligible for enrollment in this study. A CSF shunt infection was defined as identification of organisms on microbiological culture of CSF fluid obtained from a partial or complete CSF shunt system. CSF shunt system(s) included ventriculoperitoneal, ventriculoatrial, ventriculopleural, arachnoid cyst shunts, subdural shunts, and lumboperitoneal shunts; temporary devices only such as external ventricular drain(s), Ommaya reservoir(s), ventricular access devices (reservoirs) and subgaleal shunts were excluded. Negative controls included phosphate buffered saline (PBS) and five sets of both stock CSF from donors without CSF shunts. Donors included adult research participants with brain tumors, and commercially available sterile CSF from adults. Negative controls underwent concurrent deoxyribonucleic (DNA) extraction along with either one or two infection episode samples, followed by subsequent testing as described below.

### CSF Sample Collection

Sterile conditions were standard practice throughout recovery and storage of CSF. The first CSF sample for diagnosis of infection was usually obtained from needle aspiration of the shunt reservoir under sterile conditions outside the operating room in a bedside “shunt tap”. The initial CSF sample analyzed in this study either was leftover from this first diagnostic sample or was obtained in the operating room under sterile conditions from the system being removed during the first surgery to treat infection. Subsequent CSF samples, including those at the end of the infection, were generally obtained under sterile bedside conditions through a sampling port within sterile extension tubing attached to the external ventricular drain.

After CSF was obtained for the study, samples were stored at 4°C for up to a week. CSF was then aliquoted into vials of ∼100 µl for the study and stored at −70°C; PCMC samples were shipped overnight to Seattle on dry ice for analysis. After December 9, 2011, 200 µl of CSF was stored in 450 µl MO-BIO CB1 solution at the time of sample collection.

### Conventional Culture Identification of Bacteria

All CSF samples were tested using routine aerobic culture techniques in hospital-certified laboratories at both SCH and PCMC. Conventional cultures are the traditional diagnostic approach used to detect typical pathogens in infectious diseases and were performed in a clinical microbiology laboratory following Clinical and Laboratory Standards Institute guidelines; however they do not detect all bacteria present in human disease [24], [31].

### Molecular Identification of Bacteria and Fungi

For each CSF sample, bacterial DNA was extracted from either 100 µl of CSF or 200 µl of 650 µl of CSF/buffer using the BiOstic Bacteremia DNA isolation kit (MO-BIO). DNA was eluted in 100 µl of buffer. At a minimum, the initial CSF sample from the infection episode was analyzed, and in one instance the entire infection course was also analyzed.

Partial ribosomal amplification was performed as previously described. [24] The modified 16S eubacterial primers 28F, 5′-GAG TTT GAT CNT GGC TCA G-3′ and 519R, 5′-GTN TTA CNG CGG CKG CTG-3′ were used for amplifying the 500 bp region of the V1–V3 regions of the 16S ribosomal RNA (rRNA) genes. The primer sets used for FLX-Titanium amplicon pyrosequencing included the addition of linker A and an 8 base pair subject-specific barcode sequence at the 5′ end of forward primer 28F-A (5′-CCA TCT CAT CCC TGC GTG TCT CCG ACT CAG-barcode-GAG TTT GAT CNT GGC TCA G-3′) and the biotin and linker B sequence at the 5′ end of reverse primer 519R-B (5′-Biotin-CCT ATC CCC TGT GTG CCT TGG CAG TCT CAG GTN TTA CNG CGG CKG CTG-3′).

The fungal primers ITS1F, 5′- CTT GGT CAT TTA GAG GAA GTA A-3′ and ITS4R, 5′- TCC TCC GCT TAT TGA TAT GC-3′ were used for amplifying a 500 bp region of the fungal internal transcribed spacer (ITS) DNA sequence. The primer sets used for FLX-Titanium amplicon pyrosequencing included the addition of linker A and 8 base pair subject-specific barcode sequence at the 5′ end of forward primer ITS1F-A (5′-CCA TCT CAT CCC TGC GTG TCT CCG ACT CAG-barcode- CTT GGT CAT TTA GAG GAA GTA A-3′) and the biotin and linker B sequence at the 5′ end of reverse primer ITS4R-B (5′-Biotin-CCT ATC CCC TGT GTG CCT TGG CAG TCT CAG TCC TCC GCT TAT TGA TAT GC -3′).

HotStarTaq Plus Master Mix Kit (QIAGEN, CA, USA) was used for polymerase chain reaction (PCR) under the following conditions: 95°C for 5 min followed by 35 cycles of 95°C for 30 s; 54°C for 40 s and 72°C for 1 min, a final elongation step at 72°C for 10 min was also included. The PCR products were cleaned by using Diffinity Rapid Tip (Diffinity Genomics, Inc, West Henrietta, NY) and pooled. The small fragments were removed by Agencourt Ampure Beads (Beckman Coulter, CA, USA).

Tag-encoded FLX-Titanium amplicon pyrosequencing (TEFAP) was performed as described previously. [24] In preparation for FLX-Titanium sequencing (Roche, Nutley, New Jersey), a sample of double-stranded DNA was combined with DNA capture beads, and amplified by emulsion PCR. After bead recovery and bead enrichment, the bead attached DNAs were denatured with NaOH, and sequencing primers (Roche) were annealed. A 454 sequencing run was performed on a GS PicoTiterPlate using the Genome Sequencer FLX System (Roche). All FLX procedures were performed using Genome Sequencer FLX System manufacturer’s instructions (Roche). Sample analyses have been deposited in the National Institutes of Health Short Read Archive under accession number SRP031697.

### Bacterial Quantitation

*Pseudomonas aeruginosa* PAO1 (ATCC number: BAA-47) was grown in tryptic soy broth (TSB, Sigma Chemical Co., St. Louis, MO, USA) medium at 37°C with shaking for 16 hours. An aliquot was serially diluted in TSB broth. The diluted bacteria were plated out by using a Whitley automatic spiral plater (Don Whitley Scientific Ltd, Frederick, MD, USA), which was used to count colony-forming units (cfu). The bacteria broth was further diluted from 10^10^ to 10^4^ cfu ml^−1^. Total DNA extractions were performed using 1 ml of each diluted samples.

The CSF samples’ DNAs were detected using a Roche LightCycler 480. The specific primers and probe for bacteria 16S rRNA gene were as follows: 16S-F, 5′-CCA TGA AGT CGG AAT CGC TAG-3′; 16S-R, 5′-GCT TGA CGG GCG GTG T-3′; 16S-Probe, 5′-6-FAM-TAC AAG GCC CGG GAA CGT ATT CAC CG-3′. Roche LightCycler 480 Probes Master was also used for the assay. The PCR reactions were as follows: 50°C for 2 min, 95°C for 10 min, and 35 cycles of 95°C for 15 sec and 60°C for 1 min. The DNAs extracted from the series dilutions were used to set up a standard curve, the Ct values of the quantitative real-time PCR assay (qPCR) assay are compared with the standard curve. The qPCR assay using probe and primers showed that a concentration as low as 10^6^ cfu/ml can be detected on the DNA samples.

### Analysis

The proportion of individual bacterial species detected in each initial CSF sample was summarized descriptively using graphical displays. Bacterial species that comprised less than 1% of reads within a sample were grouped together as “other”. Individual species or taxa identified by sequencing were compared with findings from bacterial culture. Changes in proportion of bacterial species for a single subject over the course of CSF shunt infection treatment were tabulated for species or taxa that comprised over 2% of sequenced reads; this threshold was selected for ease of interpretation.

## Results

**Figure 1:**
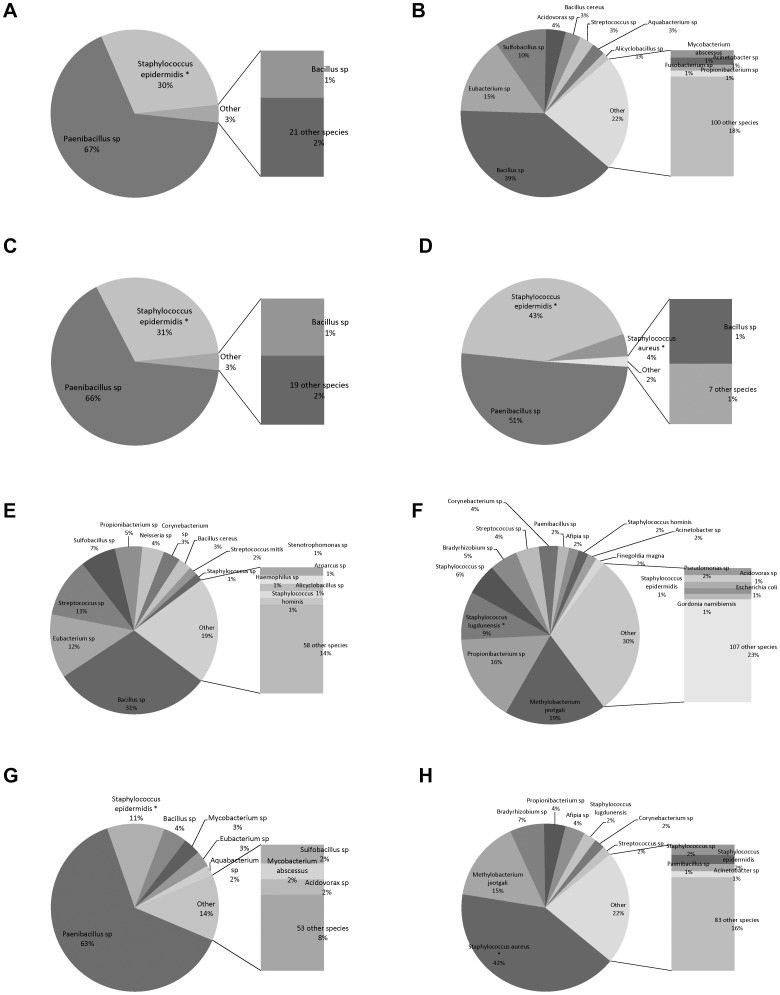
Bacterial species identified by bTEFAP at infection diagnosis. Bacterial species identified by bTEFAP in initial CSF samples from eight children with CSF shunt infection (designated “A” through “G”) are presented as a proportion of all sequence reads in the sample. Bacterial species also identified by conventional culture of the same sample are designated with an asterisk (*). Bacteria that comprised 1% or fewer of reads for each sample are designated as “other”.

Bacterial species identified by 16S rRNA bTEFAP in the initial samples from the CSF shunt infections in eight different children are shown in **Figure 1**. This sequencing method identified bacterial species that did not grow in conventional culture for all of the CSF samples. Organisms that demonstrated substantial proportions of the CSF microbiota identified by bTEFAP findings included *Paenibacillus* species, *Bacillus* species, and *Methylobacterium jeotgali*. bTEFAP of several of the samples (B, F, H) identified a large variety of additional bacterial species, each generally at relatively low abundance, and many of which are not considered typical CSF shunt infection pathogens.

Bacterial culture results are compared to 16S bTEFAP findings in **Table 1**. In all of the cases, at least one bacterial taxon was detected by both culture and bTEFAP. In six of the eight samples, the cultured microbes comprised ≥4% of the sequenced reads; in two samples (B and E), the cultured organism comprised <4% of the sequenced reads but was still present in bTEFAP. However, diverse microbes beyond those that were cultured were identified in each sample, and the cultured microbe comprised ≤0.5% of total reads in two samples. 16S bTEFAP counts from negative CSF and PBS controls processed concurrently with infection samples are also shown; negative controls demonstrated several orders of magnitude fewer bacterial reads in bTEFAP than initial CSF samples from infection episodes.

**Table 1:** Bacterial species identified by bTEFAP at infection diagnosis.

	Age at collection	Bacterial culture results	Quantitation (CFU/ml)	16S bTEFAP results	%	bTEFAP reads in patient sample/bTEFAP reads in negative control(s)	Comments
A	8 years	*Staphylococcus epidermidis*	1.15E+06	Paenibacillus sp*	67	14,950 in sample	
				*Staphylococcus epidermidis*	30	0 in control CSF	
						0 in PBS	
B	3 months	*Staphylococcus epidermidis*	<6.03E+05	Bacillus sp	39	8,369 in sample	*S.epidermidis* comprised 0.4% of bTEFAP reads
				Eubacterium sp*	15	24 in control CSF	
				Sulfobacillus sp	10	1 in PBS	
				Acidovorax sp	4		
				*Bacillus cereus* *	3		
				Streptococcus sp	3		
				Aquabacterium sp	3		
C	23 months	*Staphylococcus epidermidis*	6.28E+06	Paenibacillus sp*	66	5,694 in sample	
				*Staphylococcus epidermidis*	31	0 in control CSF	
						2 in PBS	
D	7 years	*Staphylococcus epidermidis*	3.06E+07	Paenibacillus sp*	51	5,519 in sample	
		*Staphylococcus aureus*		*Staphylococcus epidermidis*	43	24 in control CSF	
				*Staphylococcus aureus*	4	1 in PBS	
E	13 months	*Staphylococcus epidermidis*	<6.03E+05	Bacillus sp*	31	3,908 in sample	*S.epidermidis* comprised 0.5% of bTEFAP reads
				Eubacterium sp*	12	2 in control CSF	
				Streptococcus sp	11	2 in PBS	
				Sulfobacillus sp	7		
				Propionibacterium sp	5		
				Neisseria sp	4		
				Cornyebacterium sp	3		
				*Bacillus cereus* *	3		
F	8 years	*Staphylococcus lugdunensis*	<6.03E+05	*Methylobacterium jeotgali*	19	3,381 in sample	
				Propionibacterium sp	16	2 in control CSF	
				*Staphylococcus lugdunensis*	9	0 in PBS	
				Staphylococcus sp	6		
				Bradyrhizobium sp	5		
				Streptococcus sp	4		
				Cornyebacterium sp	3		
				Paenibacillus sp*	2		
G	9 months	*Staphylococcus epidermidis*	<6.03E+05	Paenibacillus sp*	63	3,820 in sample	
				*Staphylococcus epidermidis*	11	0 in control CSF	
				Bacillus sp*	5	2 in PBS	
				Mycobacterium sp	3		
				Eubacterium sp*	3		
H	2 months	*Staphylococcus aureus*	<6.03E+05	*Staphylococcus aureus*	42	3,387 in sample	
				*Methylobacterium jeotgali*	16	2 in control CSF	
				Bradyrhizobium sp	7	0 in PBS	
				Propionibacterium sp	4		
				Afipia sp	4		
				*Staphylococcus lugdunensis*	2		
				Cornyebacterium sp	2		

Results for 8 initial CSF samples from 8 subjects are reported. For the bTEFAP results, only bacteria comprising 2% or more of reads in a sample are presented, with exceptions noted in the comments column. Underlined font indicates an organism was detected with both testing methods.

indicates a bacterium is an anaerobe; there were no attempts to culture obligate anaerobes.

Fungi were also identified by ITS fTEFAP at the time of initial CSF samples for the same eight CSF shunt infections (**Table 2**). None of the CSF samples were sent for fungal cultures, so no comparison was possible. A large variety of fungi was again identified. In all cases except subjects B and D, the fTEFAP counts from negative controls were negligible compared to those from initial CSF infection samples.

**Table 2:** Fungal species identified by fTEFAP at infection diagnosis.

	Age at collection	Fungal species identified	% of reads	fTEFAP reads in patient sample/fTEFAP reads in negative control(s)
A	8 years	Cryptococcus sp	31	3,228 in sample
		Malassezia sp	24	0 in control CSF
		*Malassezia restricta*	16	
		Phaeosphaeriopsis sp	13	
		*Malassezia pachydermatis*	7	
		*Malassezia sympodialis*	4	
B	3 months	*Malassezia restricta*	37	1,802 in sample
		Wallemia sp	26	1,182 in control CSF
		Malassezia sp	14	
		Cladosporium sp	11	
		Acremonium sp Ascomycota	8	
C	23 months	*Malassezia restricta*	39	5,479 in sample
		Wallemia sp	16	0 in control CSF
		Cladosporium sp	13	
		*Rhodotorula mucilaginosa*	7	
		Aureobasidium sp	5	
		*Aspergillus penicilliodes*	5	
		Aspergillus sp	3	
		Acremonium sp Ascomycota	3	
D	7 years	*Schizophyllum commune*	23	3,919 in sample
		Cladosporium sp	18	1,182 in control CSF
		Acremonium sp Ascomycota	14	
		*Trametes elegans*	9	
		*Malassezia restricta*	8	
		Wallemia sp	7	
		*Debaryomyces hansenii*	5	
		*Malassezia sympodialis*	2	
		*Fusarium oxysporum*	2	
		Malassezia sp	2	
E	13 months	*Malassezia restricta*	20	4,779 in sample
		Sistotremastrum sp	15	93 in control CSF
		Cladosporium sp	13	
		*Didymella exitalis*	13	
		Aspergillus sp	10	
		Malassezia sp	5	
		*Malassezia globosa*	5	
		*Aspergillus penicilliodes*	4	
		*Aspergillus unguis*	3	
		Phaeosphaeriopsis sp	3	
		Wallemia sp	3	
		*Rhodotorula mucilaginosa*	3	
F	8 years	Cladosporium sp	20	3,883 in sample
		*Malassezia restricta*	14	0 in control CSF
		*Malassezia globosa*	9	
		*Emericella variecolor*	8	
		Rhinocladiella sp	8	
		*Peniophorella praetermissa*	5	
		*Spencermartinsia viticola*	4	
		*Cladosporium oxysporum*	4	
		Coprinus sp	4	
		*Cystofilobasidium capitatum*	4	
		*Malassezia sympodialis*	3	
		*Cryptococcus arrabidensis*	2	
		*Fellomyces sichuanensis*	2	
		*Malassezia pachydermatis*	2	
		*Cercospora beticola*	2	
G	9 months	*Flavodon flavus*	44	8,483 in sample
		*Antrodia xantha*	24	0 in control CSF
		*Malassezia restricta*	14	
		Eurotium sp	8	
		*Aspergillus unguis*	3	
		*Cladosporium cladosporioides*	3	
H	2 months	Cladosporium sp	37	5,266 in sample
		Wallemia sp	20	0 in control CSF
		*Cladosporium oxysporum*	6	
		*Malassezia restricta*	4	
		Ceriporia sp	4	
		*Malassezia globosa*	4	
		*Barriopsis fusca*	4	
		*Sarcomyxa serotina*	4	
		Cochliobolus sp	3	
		*Aspergillus caespitosus*	3	
		*Trichaptum larcinum*	2	

Fungal taxa identified by ITS1F-ITS4R fTEFAP in the 8 initial CSF samples that comprised at least 2% or more of reads for that sample. None of the samples was cultured for fungi.

**Figure 2:**
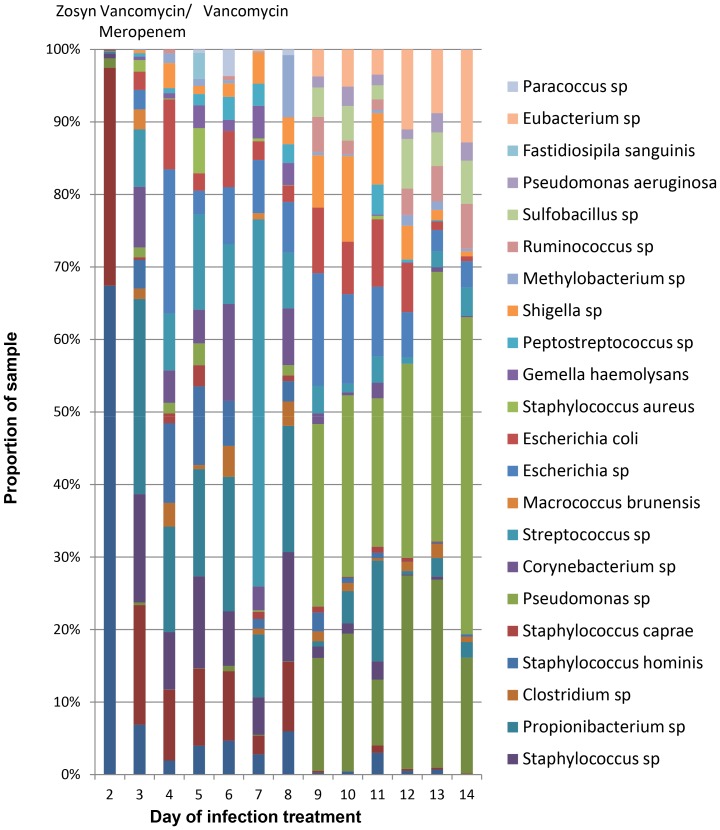
Proportion of bacterial species detected over the course of CSF shunt infection treatment by bTEFAP sequencing. Only bacterial taxa that constitute over 2% of the reads from each sample on any day are shown; fungi are not presented. No bacteria or fungi were detected in either of the two concurrent negative controls (CSF and PBS). The only CSF cultures that grew organisms was that collected on day 2, which grew *Staphylococcus epidermidis*. Surgeries were performed on day 1 (abdominal exploration), day 2 (CSF shunt removal) and day 14 (CSF shunt replacement). Antibiotic treatment provided is shown above the corresponding day it was given; for instance, vancomycin and meropenem were provided on days 2 through 5.

Finally, the proportion of bacterial species detected by bTEFAP over the course of CSF shunt infection A is shown in **Figure 2**. CSF culture on day 2 of infection identified only *S. epidermidis*, which was identified by bTEFAP along with a substantial proportion of *Paenibacillus* species. Bacterial quantitation by qPCR of the samples detected 1.15×10^7^ CFU/ml equivalents on day 2, followed by no detectable bacterial DNA above the assay’s limits of detection (<6×10^5^ CFU/mL equivalents) thereafter. After day 2, bTEFAP identified a large variety of other species at relatively low abundances throughout the course of infection treatment. bTEFAP did not identify any 16S reads among CSF and PBS negative controls. The proportions of some bacteria fluctuated, such as *Streptococcus* species; others, such as *Bacillus* and *Pseudomonas* species, which are not targets of antimicrobial therapy, even rose over the course of infection treatment. Intravenous antibiotics that were used in this infection and that may have driven the observed microbial abundance changes are shown in **Figure 2**. Similarly, CSF shunt surgeries, which could also have impacted the microbial ecology, were performed on day 2 (shunt removal) and 14 (new shunt placement) in infection A; samples after day 2 were obtained regularly using sterile technique from an external ventricular drain placed in the ventricle.

## Discussion

To our knowledge, this is the first study that describes the microbiota present in CSF shunt infection. Using 16S and ITS TEFAP, we have demonstrated that numerous bacterial species, including anaerobes, as well as fungal organisms were detected in the CSF of children with CSF shunt infections. With the exception of fungal counts in two samples, these organisms were detected at TEFAP counts well above those in negative controls. The organisms recovered by TEFAP, including many beyond those recovered by standard aerobic bacterial culture, suggest heterogeneous microbiota. The composition of the microbiota changed over the course of infection treatment, probably in response to antibiotic use and surgical removal of the shunt. Future studies of a larger patient population will be needed to determine the relationships between the diverse CSF microbiota and clinical outcomes, particularly the response to treatment and occurrence of reinfection.

Bacterial organisms that comprised substantial proportions of the 16S TEFAP findings included *Paenibacillus* species, *Bacillus* species, and *Methylobacterium jeotgali*. *Paenibacillus* species are particularly intriguing in this context, as they have been reported to have biomedical applications. [32] Likewise, several anaerobic bacteria were identified by bTEFAP, as well as fungal organisms by fTEFAP. None of these three organisms are considered typical CSF shunt infection bacterial pathogens, and the clinical relevance of the identification of these additional bacterial and fungal organisms beyond conventional aerobic culture is not yet clear. However, these findings suggest that clinicians should consider anaerobic bacterial and fungal cultures in addition to standard aerobic bacterial culture in initial evaluation of CSF shunt infection. Our results further indicate that culture-independent approaches may have utility in the diagnosis of CSF shunt infections and in directing their treatment.

16S bTEFAP analysis suggested that CSF bacterial microbiota changed over the course of infection treatment. The results in **Figure 2** suggest that these changes occur due to therapy, which included the standard treatment with surgery and intravenous antibiotics targeting the organism recovered from culture. While the clinical relevance of this changing microbiota composition also requires further investigation, the continued detection of diverse bacteria during the course of treatment shown in **Figure 2** indicates that microbial persistence within the CSF despite standard treatment could be a source for the recurrence of CSF shunt infections, a frequent and troubling occurrence. In this study cohort, one child (D) to date has developed re-infection to date with *Staphylococcus aureus*; however, the proportion or burden of *S. aureus* at the time of shunt replacement is unknown. Study of additional, longitudinal samples from children who have developed serial CSF shunt infections is needed to determine if bacterial species identified at the first infection are related to those seen at the second infection. Further investigations such as these will hopefully inform more effective CSF shunt infection treatment.

There are several limitations to this work. The selection of appropriate negative controls is difficult. Since the negative controls we selected were from adults, they may not be physiologically comparable to the infection samples. While we considered the use of CSF from children with shunt obstruction without clinical evidence of infection, we were concerned about the potential role of microbial biofilms in this clinical scenario and felt it warrants a separate, independent line of inquiry. CSF from children with functioning shunts may be an ideal negative control, but challenging to obtain. Moreover, while the ideal negative control would be CSF from uninfected children whose shunts have been in-situ for a similar period to the infected cases, this would not be ethically feasible since sampling of CSF from the shunt introduces risk of infection. We anticipate future work examining bacteria and fungal sequences will need to incorporate negative controls from several sources, including but not limited to: donor CSF, children undergoing shunt revision surgery for shunt failure, and children undergoing initial CSF shunt placement.

In addition, there are several opportunities for contamination of CSF samples during recovery, storage, and experimentation. However, sterile conditions were sought throughout recovery and storage of CSF samples, and contamination during experimentation is improbable given low recovery from negative controls that were handled identically to and concurrently with the CSF samples. Therefore, contamination is unlikely to have a major contribution to the bacterial and fungal diversity observed. The low numbers of infection episodes analyzed renders this study preliminary in nature. Testing from the hospital-certified laboratories was limited to routine aerobic cultures, practices that are subject to individual laboratory practices, including policies affecting identification of all organisms on a plate, incubation for extended periods of time, and enrichment for slow growing and fastidious organisms. We did not have information about variability or density of colonies on the original plates. In addition, fungal and anaerobic cultures were not obtained. Finally, detection of microbial DNA, which could be from either viable or nonviable cells, by PCR does not guarantee that living bacteria or fungi were present at the time of sample collection. However, the differences observed between infection and control samples indicates that these microbes were present at some point up to collection.

While the clinical importance of these CSF microbiota remains to be determined, the current findings provide additional support to the emerging evidence of a role for biofilms in CSF shunt infection. Biofilms are often comprised of multiple microbial taxa held together by an extracellular matrix, [25] and they frequently persist despite antibiotic treatment. This model is consistent with our concurrent detection of diverse microbes in each sample, and the persistence of much of this microbiota during antimicrobial therapy. Additional studies of cell pellets from the CSF, the shunt material and tubing itself, as well as studies of animal models and modes of infection, may shed light on the importance of biofilms for these findings.

## Conclusions

Numerous bacterial and fungal organisms appear to be present in the microbiota that comprises CSF shunt infection. In evaluating CSF shunt infection, fungal and anaerobic bacterial cultures should be considered in addition to aerobic bacterial cultures, and culture-independent approaches may prove to be useful as a primary or adjunctive diagnostic modality. Furthermore, better understanding of the diverse microbial environment in active CSF shunt infection is needed. The persistence of these microbiota despite antibiotics may suggest the presence of biofilm. More effective treatment of CSF shunt infection is badly needed to reduce unacceptably high rates of reinfection, and this early work suggests that one effective target may be the reduction of the diverse microbiota present in infection. Future work must focus on defining the relationships between the diverse CSF microbiota found in this study with clinical outcomes, including response to treatment and reinfection; as well as on attainment of optimal negative control subjects.
